# Next‐generation Alzheimer's therapeutics: target assessment and enablement at the Indiana University School of Medicine–Purdue University TREAT‐AD Center

**DOI:** 10.1002/alz.70964

**Published:** 2026-01-13

**Authors:** Timothy I. Richardson, Rebecca C. Klein, Kun Huang, Jie Zhang, Andrew D. Mesecar, Jeffrey L. Dage, Brent Clayton, Bruce T. Lamb, Alan D. Palkowitz

**Affiliations:** ^1^ Department of Medicine, Division of Clinical Pharmacology Indiana University School of Medicine Indianapolis Indiana USA; ^2^ Stark Neurosciences Research Institute Indiana University School of Medicine Indianapolis Indiana USA; ^3^ Indiana Biosciences Research Institute Indianapolis Indiana USA; ^4^ Regenstrief Institute Indianapolis Indiana USA; ^5^ Department of Medical and Molecular Genetics Indiana University School of Medicine Indianapolis Indiana USA; ^6^ Department of Biochemistry Purdue University West Lafayette Indiana USA; ^7^ Department of Neurology Indiana University School of Medicine Indianapolis Indiana USA

**Keywords:** biochemistry, bioinformatics, drug discovery, INPP5D, medicinal chemistry, pharmacology, PLCG2, project management, SHIP1, structural biology

## Abstract

The incidence of Alzheimer's disease (AD) continues to increase, despite decades of effort to develop disease‐modifying therapies. In response, the National Institute on Aging (NIA) established the TaRget Enablement to Accelerate Therapy Development for Alzheimer's Disease (TREAT‐AD) centers to address the gap between basic research and translational drug discovery. Situated within a robust AD research environment, the Indiana University School of Medicine (IUSM)–Purdue University TREAT‐AD Center is one of two National Institutes of Health (NIH)‐supported centers funded to accomplish this mission. With a focus on novel biological targets beyond amyloid and tau, our center has assembled the necessary components of a drug discovery engine: project and data management, bioinformatics and computational science, structural biology and biochemistry, assay development and pharmacology, and molecular design and synthesis of small molecules, antibodies, and oligonucleotides. Our objective is to deliver Target Enabling Packages (TEPs) within an open science framework, making data, methods, and research tools broadly accessible through the AD Knowledge Portal.

**Highlights:**

- The Indiana University School of Medicine (IUSM)–Purdue TREAT‐AD Center develops Target Enabling Packages (TEPs) to advance novel targets for the treatment of Alzheimer's disease (AD).
- The center is overseen by an administrative core and operates through four technical cores – bioinformatics, structural biology, assay development, and medicinal chemistry – within a milestone‐driven and open science framework.
- Multi‐omics, systems biology, and machine learning (ML) approaches guide the nomination of high‐priority targets beyond amyloid and tau.
- Cross‐core workflows provide structural insights into novel biological targets, validated assays, biomarkers, and molecular probes that enable lead optimization.
- All data, methods, and tools are openly shared through the AD Knowledge Portal to accelerate global efforts in AD drug discovery.

## 1 INTRODUCTION

Alzheimer's disease (AD) and related dementias (ADRD) are progressive neurodegenerative disorders affecting 7.2 million Americans, which is a number that will grow to over 14 million by 2060 as the number of adults age 65 and older increases.^1^, ^2^ The medical and economic burden of ADRD on individuals and society is well documented.^3^ Although the amyloid beta (Aβ) hypothesis has guided AD research for 30 years,^4^, ^5^ amyloid‐targeted therapies have been challenging to develop, and amyloid removal provides only minimal slowing of cognitive decline.^6^ In 2014, the National Institute on Aging (NIA) began supporting research programs funded to explore the complex interactions between human genetics and disease through the Accelerating Medicines Partnership (AMP‐AD).^7^ In 2019, NIA launched TaRget Enablement to Accelerate Therapy Development for Alzheimer's Disease (TREAT‐AD) centers to advance the translational aspects of this research by bridging gaps to novel therapeutic development, the so‐called “valley of death” between basic research and clinical studies. The centers build directly on the work of the AMP‐AD program, which has identified over 500 candidate targets. Built on open‐science principles, the centers aim to advance these novel targets through collaborative efforts to develop capabilities and tools for drug discovery programs.

The IUSM–Purdue University TREAT‐AD Center is one of two National Institutes of Health (NIH)‐supported centers funded to support this mission, along with the Emory‐Sage‐Structural Genomics Consortium (SGC)‐Jackson Laboratory (JAX) TREAT‐AD Center. Our center is focused on developing high‐quality research tools necessary to enable a portfolio of biological targets with all the necessary capabilities to optimize lead molecules as candidates for clinical development. Leveraging advanced multi‐omics analytics, bioinformatics, structural biology, assay development, screening, and medicinal chemistry, the center produces comprehensive target enabling packages (TEPs) that include target proteins, validated assays, pharmacodynamic biomarkers, and chemical probes. The data, methodologies, pharmacological tools, and chemical probes generated by the center are openly and freely shared with the global research community through the AD Knowledge Portal.^8^ This perspectives article outlines the center's scientific rationale, operational model, and contributions to the NIA's effort to enable the next generation of AD therapeutics through translational research and open science.

Our center is supported by the long‐standing institutional commitment of Indiana University to the study of AD through the Stark Neurosciences Research Institute and NIA‐supported initiatives, including the Indiana Alzheimer's Disease Research Center founded in 1991, the National Centralized Repository for Alzheimer's Disease (NCRAD), the Model Organism Development and Evaluation for Late‐onset Alzheimer's Disease (MODEL‐AD) Consortium, and the Longitudinal Early‐Onset Alzheimer's Disease Study (LEADS). The added partnerships of Purdue University, the University of Pittsburgh School of Medicine, and the Indiana Biosciences Research Institute (IBRI) maximize scientific engagement between world‐class scientists, connecting them to institutions focused on both AD research and drug discovery.

**FIGURE 1:**
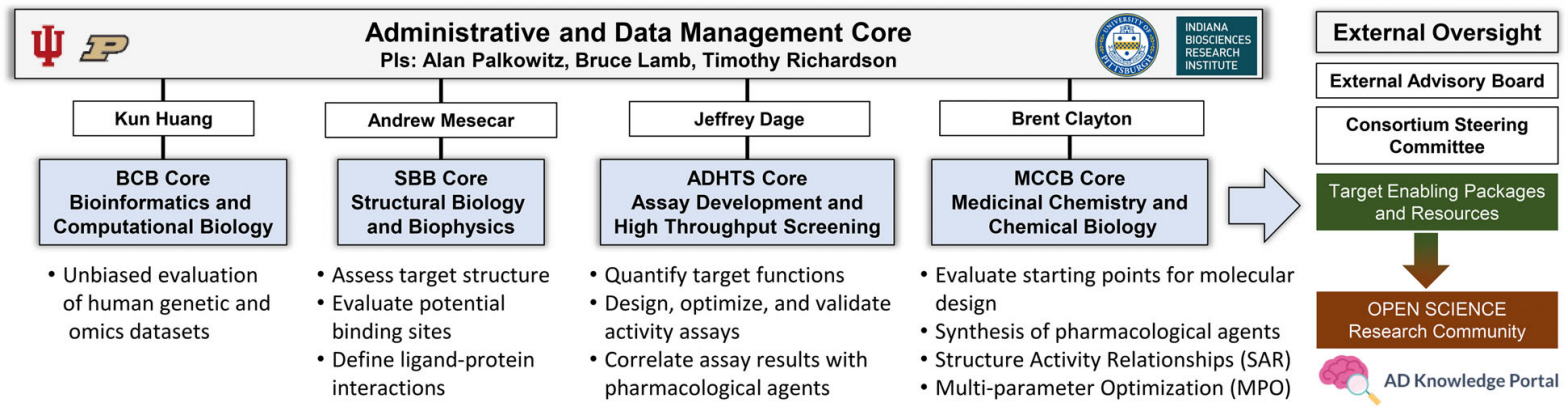
IUSM–Purdue TREAT‐AD Center Organizational Structure. IUSM, Indiana University School of Medicine; TREAT‐AD, TaRget Enablement to Accelerate Therapy Development for Alzheimer's Disease.

The center is organized into four highly integrated and collaborative technical cores that are managed through a common Administrative and Data Management Core (Figure 1). The technical cores include Bioinformatics and Computational Biology (BCB), Structural Biology and Biophysics (SBB), Assay Development and High‐Throughput Screening (ADHTS), and Medicinal Chemistry and Chemical Biology (MCCB). To complement our capabilities, we have established a network of external resources and collaborators that increase capacity and access additional expertise. Target Development Teams co‐led by biology and chemistry project leaders ensure accountability for milestone achievement and provide the necessary focus and scientific expertise for decision‐making in response to data.

## 2 METHODS

While human genetics and multi‐omics datasets provide a foundation for target nomination, prioritization requires nuanced scientific judgment from each technical core. In practice, this process integrates evidence from human biology, preclinical models, and the existing literature to assess both biological target validation and practical considerations related to modality choice, druggability, and translational feasibility. For each target, we evaluate (1) the strength and reproducibility of genetic and transcriptomic associations, including effect size, directionality, and consistency across independent datasets, while documenting conflicting or uncertain findings; (2) published results in cellular and animal models, particularly when reported results are inconsistent or phenotypes raise safety concerns (e.g., knockout lethality); and (3) whether prior efforts failed due to target biology in an AD‐relevant context, the quality of the pharmacological agents used to test the therapeutic hypothesis, or the selection of preclinical models and biomarker strategies used to assess outcomes.

### 2.1 Administrative and Data Management (ADMIN) Core

The ADMIN Core plays a central role in ensuring that the center achieves its overarching strategic mission, project‐specific aims, and milestones. It oversees the administration of the center and the integration of the technical cores while maintaining regular communication with and implementing recommendations from both the center steering committee (CSC) and an external advisory board (EAB). The CSC is made up of center codirectors, technical core leads, collaborative principal investigators (PIs), NIH scientists, and the NIA program officers overseeing milestone deliverables. The EAB is made up of key opinion leaders in the fields of AD research and drug discovery. The EAB meets once a year to provide independent feedback to the CSC on the center's performance, project priorities, progress, and any strategic or tactical changes. The ADMIN Core provides leadership and oversight across several critical areas, including the management and prioritization of the center's portfolio of projects, the development and execution of technical strategies, and the seamless integration of each technical core's activities. Additionally, it is responsible for identifying and resolving project bottlenecks, making dynamic resource decisions, and implementing alternative strategies when necessary to ensure success. The ADMIN Core also conducts technical reviews and provides recommendations for milestone‐based go/no‐go decisions. A key responsibility includes oversight of TEPs and ensuring associated datasets are submitted to the AD Knowledge Portal.

### 2.2 BCB Core

The BCB Core supports the center's drug discovery efforts by (1) advancing target selection through the integration and analysis of diverse genetic and omics datasets using bioinformatics, systems biology, machine learning (ML), and statistical methods and (2) collaborating with the other cores on developing and applying advanced bioinformatics and artificial intelligence (AI) methods for tasks such as biomarker discovery and lead molecule prediction. In recent years, advancements in single‐cell omics technology, the accumulation of single‐cell data, and the integration of AI/ML techniques have led to new insights in AD research. The BCB Core, therefore, leverages ML for generating new target hypotheses from both bulk tissue and single‐cell data and integrates them with other data modalities. In addition, the BCB Core is developing novel three‐dimensional graph‐based learning methods to generate small‐molecule structures, further enabling computational support for therapeutic development. From there and throughout the enablement process, the BCB Core provides support to the other technical cores, including general bioinformatics and biostatistics for study design, data processing, and analysis.

### 2.3 SBB Core

The SBB Core provides a comprehensive gene‐to‐structure platform to support the selection and advancement of prioritized therapeutic targets. It generates protein constructs for use in assay development, validation, and structural studies, employing biochemical and biophysical methods to assess the kinetics and thermodynamics of ligand–protein interactions. The structures of target proteins with bound ligands obtained with X‐ray crystallography and cryogenic electron microscopy (cryo‐EM) combined with thermodynamic analyses to characterize compound–protein interactions provide insights into ligand–protein interactions and subsequent modulation of activity. This multifaceted approach to protein analysis and drug design includes desorption electrospray ionization mass spectrometry (DESI‐MS), utilized to create novel, label‐free biochemical assays for challenging target proteins, XtalPred3 for predicting protein crystallizability and generating protein constructs, followed by automated protein crystallization and imaging that are enhanced through the use of AIDA4, AIDA5, and AlphaFold models to accelerate model building. With these approaches the SBB Core conducts in‐depth enzymology and mode of action (MOA) studies to elucidate the biochemical mechanisms underlying compound activity, helping to guide translation from biochemical assays to cellular models.

### 2.4 ADHTS Core

The ADHTS Core plays a critical role in enabling nominated biological targets by establishing a mechanistic hypothesis that guides the development of robust, interconnected in vitro assay systems. These assays generate high‐quality data to support structure–activity relationship (SAR) studies performed by the MCCB Core and lead to in vivo pharmacokinetics (PK)/pharmacodynamics (PD) characterization and efficacy studies. Target engagement (TE) and PD biomarkers that translate from preclinical models to human studies dramatically increase the success and quality of clinical research. Therefore, the core also works to identify translational biomarkers for each target and mechanism utilizing in vitro and in vivo assays while integrating translational assays designed to operate in a clinical setting to inform human studies.

### 2.5 MCCB Core

The MCCB Core serves as a key nexus point of the center, integrating data from all technical cores to drive the discovery, design, synthesis, and delivery of high‐quality pharmacological tools for target validation. It evaluates target druggability and selects the most suitable therapeutic modality, including small molecules, antibodies, and oligonucleotides. The core employs diverse ligand identification strategies to uncover novel molecular starting points and conducts SAR studies using multi‐parameter optimization (MPO) to refine both pharmacological activity and drug‐like properties. An iterative design‐make‐test‐analyze methodology enables optimization of multiple parameters simultaneously with input from each technical core. A suite of computational capabilities, including a drug discovery workflow and data management system, is coupled to an informatics platform and molecular modeling software that includes calculated physicochemical parameters and properties related to absorption, distribution, metabolism, and excretion (ADME). These capabilities, along with powerful computational resources available through Indiana University, drive the design of ligands with better affinity and potency while maintaining drug‐like properties. In collaboration with the BCB Core, the MCCB Core utilizes novel AI‐based molecular design pipelines incorporating cheminformatics databases, protein 3D structures, and molecular structure to virtually screen and predict small molecules for biological targets. With a flexible approach to medicinal chemistry, the MCCB Core advances a broad portfolio of therapeutic modalities against prioritized targets and provides the research community with well‐characterized chemical probes suitable for further lead development.

### 2.6 Project management and milestones

Drug discovery and development requires expertise spanning a wide range of scientific disciplines. Our center is led by individuals who have a deep understanding of AD biology and extensive drug discovery and clinical development experience. This broad experience is coupled with deep expertise in data science, protein biochemistry and biophysics, pharmacology, and chemistry. As an academic drug discovery center, we also have the important objective of training the next generation of AD researchers. Through the experience of preclinical research within a center focused on delivering high‐quality enabling packages, we also seek to provide high‐quality training in the integrative science of drug discovery through postdoctoral fellowships, graduate student projects, and collaborations with other training programs.

**FIGURE 2:**
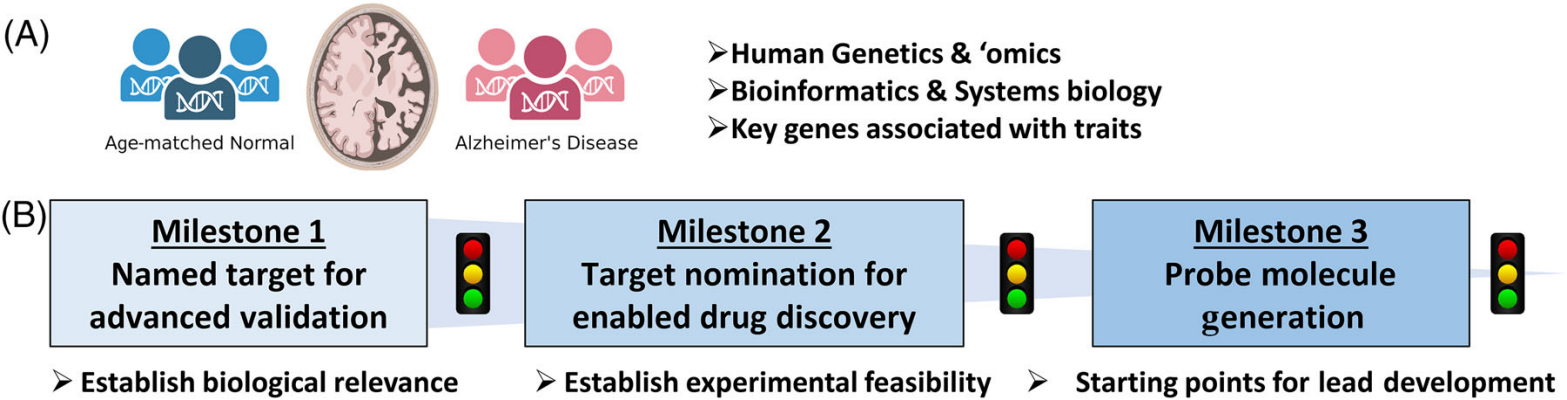
Milestone‐driven pipeline for Alzheimer's drug target selection and enablement. (A) The target nomination and validation workflow begin with comparative analysis of human genetics and multi‐omics datasets from AD patients and age‐matched normal controls to identify potential therapeutic targets. (B) Targets selected for Milestone 1 undergo a rigorous evaluation of biological validation and druggable tractability. Milestone 2 assesses the feasibility of initiating a drug discovery program, including evaluation of existing ligands and pharmacological assays. Milestone 3 involves intensive medicinal chemistry and assay development efforts focused on generating high‐quality probe molecules that fulfill the early‐stage criteria set by the NIA's Alzheimer's Disease Drug Development Program. AD, Alzheimer's disease; NIA, National Institute on Aging.

The center's capabilities are organized to efficiently nominate targets and enable them for a drug discovery program. We have established clearly defined milestones and implemented a project management function within the center to align all efforts toward achieving our goals (Figure 2). Human genetics and the unbiased evaluation of omics datasets form the foundation of target nomination. Bioinformatics and systems biology approaches are used to identify key genes associated with AD traits in comparison to age‐matched controls. Milestone 1 assesses biological relevance by evaluating the literature and assessing the current state of knowledge regarding target validation and druggability. Most of the hundreds of potential targets considered do not meet the criteria for biological relevance and druggability defined by each technical core, or a mechanistic hypothesis cannot be established. However, when the technical cores of the center do align on the *potential* feasibility of a target with a mechanistic hypothesis, Milestone 2 determines the *experimental* feasibility of enabling a drug discovery program by evaluating and establishing initial in vitro assays and tool molecules. Often, we find that reported results cannot be reproduced, the molecules are inadequate, or the assays are unsuitable in a biological context relevant to AD. In some cases, a drug target has already been sufficiently enabled, and in these instances, we provide guidance on the best tools for AD research. Targets deemed to have a sufficient probability of success and significant potential for innovation are advanced to Milestone 3. This final milestone requires the greatest investment of time and resources, with the goal of delivering a lead molecule series that meets the early‐stage entry criteria of the Alzheimer's Drug Development Program (ADDP) established by the NIA.

## 3 TARGET ENABLEMENT

### 3.1 Target selection

**FIGURE 3:**
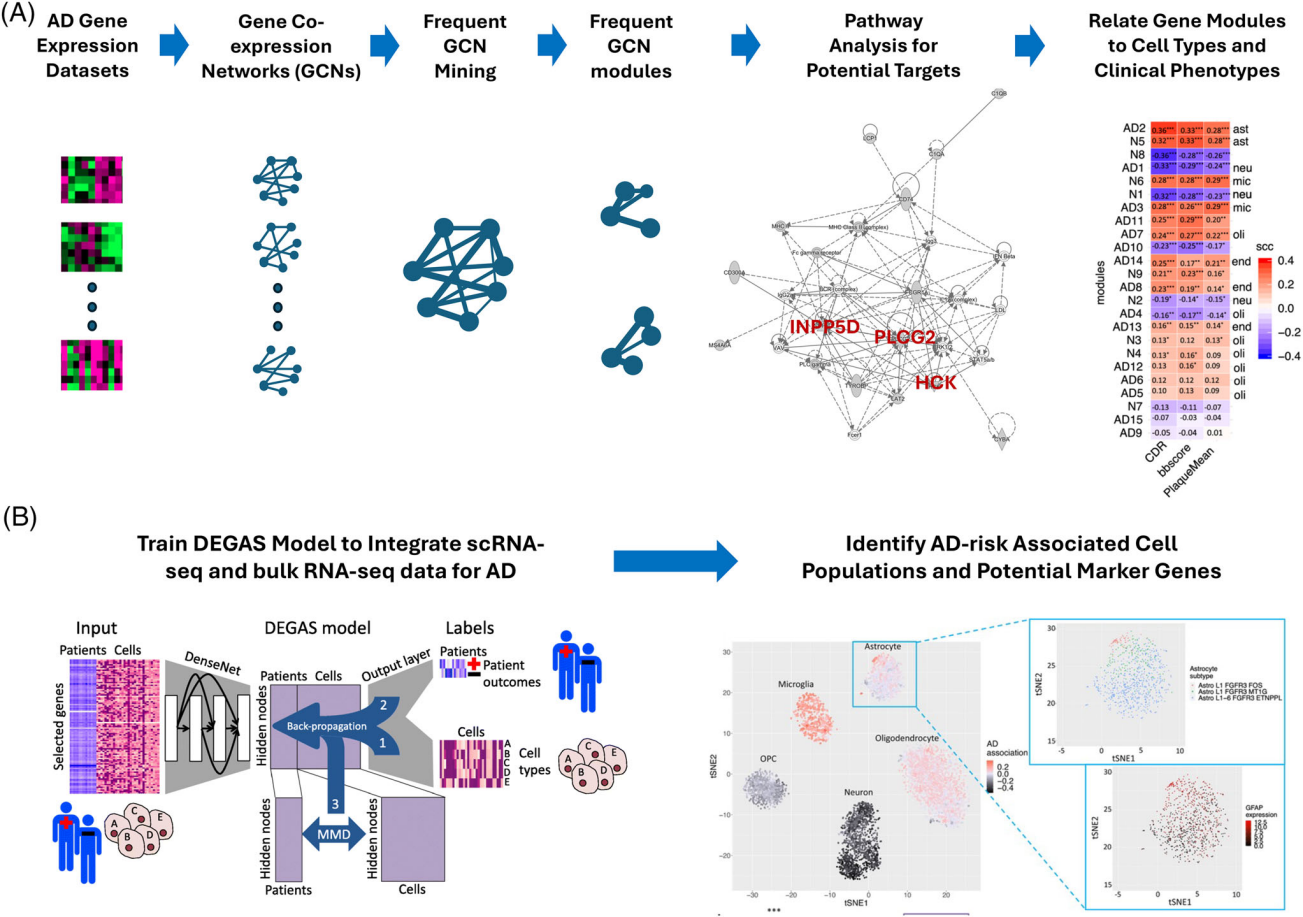
BCB Core strategy for target selection. (A) Integrating multiple large gene transcriptome (RNA‐seq) datasets to mine frequent GCNs and identify frequent GCN modules that are associated with different cell types and clinical phenotypes. (B) Integrating scRNA‐seq and bulk RNA‐seq datasets to identify AD‐associated cell populations and their marker genes as well as potential targets. AD, Alzheimer's disease; BCB, Bioinformatics and Computational Biology; GCN, gene co‐expression network; scRNA‐seq, single‐cell RNA‐seq.

By supporting research consortia beginning with AMP‐AD, the NIA has recognized the value of human genetics in establishing the next generation of AD targets. Historically, research has focused on the hallmarks of the disease: amyloid and tau. Beyond these hallmarks is the observation that some individuals are resistant to neurodegeneration regardless of amyloid and tau levels, while others are susceptible due to a combination of genetic and environmental factors. Moreover, using human genetics as the basis for target selection increases the probability of clinical success across therapeutic areas.^9^ The strategy of the BCB Core is built on this foundation and follows the processes outlined in Figure 3.

Initially, the focus is on human *post mortem* brain bulk tissue transcriptomics to identify potential AD‐associated signature genes and pathways. By applying a gene co‐expression network analysis and module mining developed by PIs within the BCB Core,^10^, ^11^ a set of co‐expressed gene expression modules is identified that play important roles in the disease. AD‐associated frequent gene co‐expression network analysis (fGCN) modules were identified across eight large transcriptomic cohort studies from multiple regions of human *post mortem* brain samples, which bear unique AD signature genes and are highly enriched with specific brain cell type markers, differentially expressed genes, and known AD risk genes. The identified microglia and astrocyte modules were chosen for further study with a focus on AD‐related neuroinflammation and the phagocytotic functions of microglia. Through protein–protein interaction (PPI) network analyses, one AD‐related microglia module was found to be centered on the gene INPP5D, which encodes SHIP1, and has been shown to regulate microglial functions. The INPP5D‐tightly‐associated gene PLCG2 was also identified within that module. Due to their key roles in immune signaling in AD‐associated microglia, SHIP1 and PLCG2 were selected as a potential drug targets for further evaluation.

With the accumulation of a large amount of single‐cell RNA‐sequencing (scRNA‐seq) from brain tissues, especially from longitudinal cohort studies such as the Religious Orders Study (ROS) and the Memory and Aging Project (MAP), it became imperative to integrate both bulk and single‐cell omics data for target discovery. The BCB Core has developed new methods for integrating scRNA‐seq and bulk tissue RNA‐seq data with the clinical annotations for samples and cells. Specifically, the Diagnostic Evidence GAuge of Single cells (DEGAS) framework was developed using a multi‐task learning approach based on deep neural networks.^12^ It can “transfer” the sample level clinical annotations (e.g., AD vs normal) to individual cells and identify high‐risk cell populations, which can be used to discover new markers and target genes.

### 3.2 Target engagement

**FIGURE 4:**
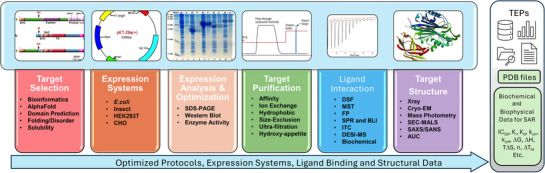
SBB Core strategy for target engagement and structural studies. Integrated workflow for target selection, protein expression, purification, ligand interaction studies, and structural determination. These approaches generate high‐quality biochemical, biophysical, and structural data that support TEPs and provide a foundation for SAR studies. SAR, structure‐activity relationship; SBB, structural biology and biophysics; TEPs, Target Enabling Packages.

In order for a drug‐like molecule to serve as a useful pharmacological tool for target validation and as a starting point for molecular optimization, it must engage its intended target. The most straightforward approaches for assessing TE are typically conducted in vitro with purified target proteins and designed compounds. The overall strategy of the SBB Core is illustrated in Figure 4, which ensures that the center achieves this goal from the very beginning.

The crucial first step in the process is to ascertain whether a target protein of interest has the potential for being produced in sufficient quantities for biochemical and biophysical assays as well as structural studies. The “target selection” process starts by using bioinformatics tools^13^, ^14^, ^15^, ^16^, ^17^ and available literature to determine whether the target protein can be expressed and purified either intact with all of its known or predicted domains or whether it will have to be dissected and produced as individual domains either alone or in combination. A number of target proteins identified via bioinformatics can fail at this stage, having too many intrinsically disordered regions or transmembrane domains. If a target protein is predicted to have a high probability of being stable with soluble domains, we select expression systems to produce the protein in high purity. The first choice is *Escherichia coli* or insect cells because of their ease of use and lower associated costs. If these two expression systems are unsuccessful, then mammalian systems, either HEK293 or CHO cells, would be pursued. Target proteins are purified using different approaches depending on the affinity tag utilized for protein expression and predicted protein properties such as molecular weight and isoelectric point. At least two steps, affinity, ion exchange, and/or size exclusion, are needed to produce protein pure enough and in sufficient quantities for TE studies.

There are multiple experimental approaches for determining whether a ligand engages its protein target in vitro. If the target is an enzyme, then the first choice is typically modulation of its enzymatic activity by the ligand using a convenient enzyme activity assay. In addition, enzyme assays typically use very little protein, which is an advantage. Quantitative values for enzyme inhibition or activation, IC_50_ and EC_50_, are obtained to support the SAR studies that drive ligand optimization. For some enzyme targets, however, convenient in vitro enzymatic assays are not available. Additionally, most potential targets in the human genome are not enzymes, and therefore TE studies require biophysical approaches.

The most straightforward biophysical approach to evaluate whether a ligand binds to the protein of interest is to determine whether the melting temperature (Tm) of the protein changes in the presence of the ligand. A change in a protein's Tm value can indicate whether a small molecule stabilizes (+ΔTm) or destabilizes (−ΔTm) the protein upon binding. Differential scanning fluorescence (DSF) is a readily available, qualitative approach to determining protein Tm values. DSF studies are performed using an reverse transcription polymerase chain reaction machine and protein‐binding dyes such as SYPRO‐orange, GlowMELT, or 8‐anilinonaphthalene‐1‐sulfonic acid (ANS). DSF using intrinsic protein fluorescence, also known as nano‐DSF, can also be used as a label‐free approach to determine shifts in protein Tm values. Other fluorescence approaches, such as microscale thermophoresis (MST) and fluorescence polarization (FP), are often used and provide quantitative assessments that determine the dissociation constants (*K*_d_ values) of ligands binding to their target proteins. Both MST and FP require specialized instrumentation, and each has its own experimental merits and limitations. MST can utilize proteins labeled with dye molecules or protein intrinsic fluorescence, and FP requires a fluorescently labeled molecule already known to bind to the protein of interest.

Surface plasmon resonance (SPR) and biolayer interferometry (BLI) are two label‐free biophysical approaches that can be used to determine the kinetics of ligand binding to a target protein. The protein of interest is attached to a biosensor either chemically or through affinity binding, and the association or “on” rates (*k*_on_) and dissociation or “off” (*k*_off_) rates are measured. From these values, equilibrium dissociation constant (*K*_d_) values can be determined for compounds, and together with the *k*_on_ and *k*_off_ values, compound series SARs can be established. Isothermal titration calorimetry (ITC) is a label‐free and immobilization‐free method that is used to determine the binding enthalpy (ΔH), binding affinity (ΔG), equilibrium dissociation constant (*K*_d_), binding entropy (TΔS), and stoichiometry (n) of protein–ligand interactions. The determination of thermodynamic constants for protein–ligand interactions can help determine which groups on the compound drive binding potency, aiding in structure‐based drug design (SBDD) efforts.

When it comes to utilizing biochemical and biophysical approaches for assessing TE, our general approach is to evaluate all amenable approaches for a target to establish which ones work best so that we can establish an efficient and robust workflow for the evaluation of a large number of compounds. We have found that each target protein, or individual domain(s) of a target protein, is only amenable to some of the approaches described earlier. Ultimately, it is the combination of biochemical and biophysical studies that yields the best success.

Finally, the SBB core is engaged in the determination of the X‐ray and/or cryo‐EM structures of the target proteins in their unbound and ligand‐bound states. The goal is to achieve structural determination at better than 3 Å resolution, which provides accurate models for computational modeling and SBDD. The program XtalPred^17^ is used to help predict the crystallization potential for target proteins, and high‐throughput crystallization approaches are utilized for each target. The oligomeric or quaternary structures of all protein constructs are determined by mass photometry (MP) and size exclusion chromatography‐multi‐angle light scattering (SEC‐MALS), and quaternary structure arrangements are checked by modeling using AlphaFold multimer.^18^ Dynamic light scattering (DLS) and static light scattering (SLS) are also used to evaluate protein aggregation and polydispersity to aid in identifying buffer conditions that stabilize the protein with high monodispersity for protein crystallization and cryo‐EM grid preparation.

Ultimately, the optimized protocols for expression, purification, and characterization of each target protein are provided by the SBB Core to the scientific community through TEPs and peer‐reviewed literature. Ligand binding data, for example, kinetic and thermodynamic constants, are also provided in TEPs and the open literature. Expression vectors for each protein are made available through Addgene, and protein structures are deposited into the Protein Data Bank (PDB).

### 3.3 Target assessment

Beyond the biochemical and biophysical assays described above, a ligand must not only engage its target but also modulate it in a way that produces the desired biological effect. Pharmacology measures how molecules interact with biological systems in a concentration‐dependent manner that can be quantified. In this way, it serves as a bridge between the biology that drives disease and the chemistry required to optimize therapeutic agents. First, the measured biological effects need to connect to a hypothesis on how it will modify disease onset or course. The ADHTS Core works with the BCB Core to establish a clear mechanistic hypothesis and then determines whether a prioritized target is amenable for cell‐based assays either for high‐throughput screening (HTS) or SAR studies related to mechanism. HTS and SAR assay development criteria are unique to each target, but both must start with an understanding of the biology and a target's potential roles in the disease process. In parallel to the in vitro assessment, we perform basic assessments of available in vivo models to explore the relevance of a target through its proposed mechanism of action.

**FIGURE 5:**
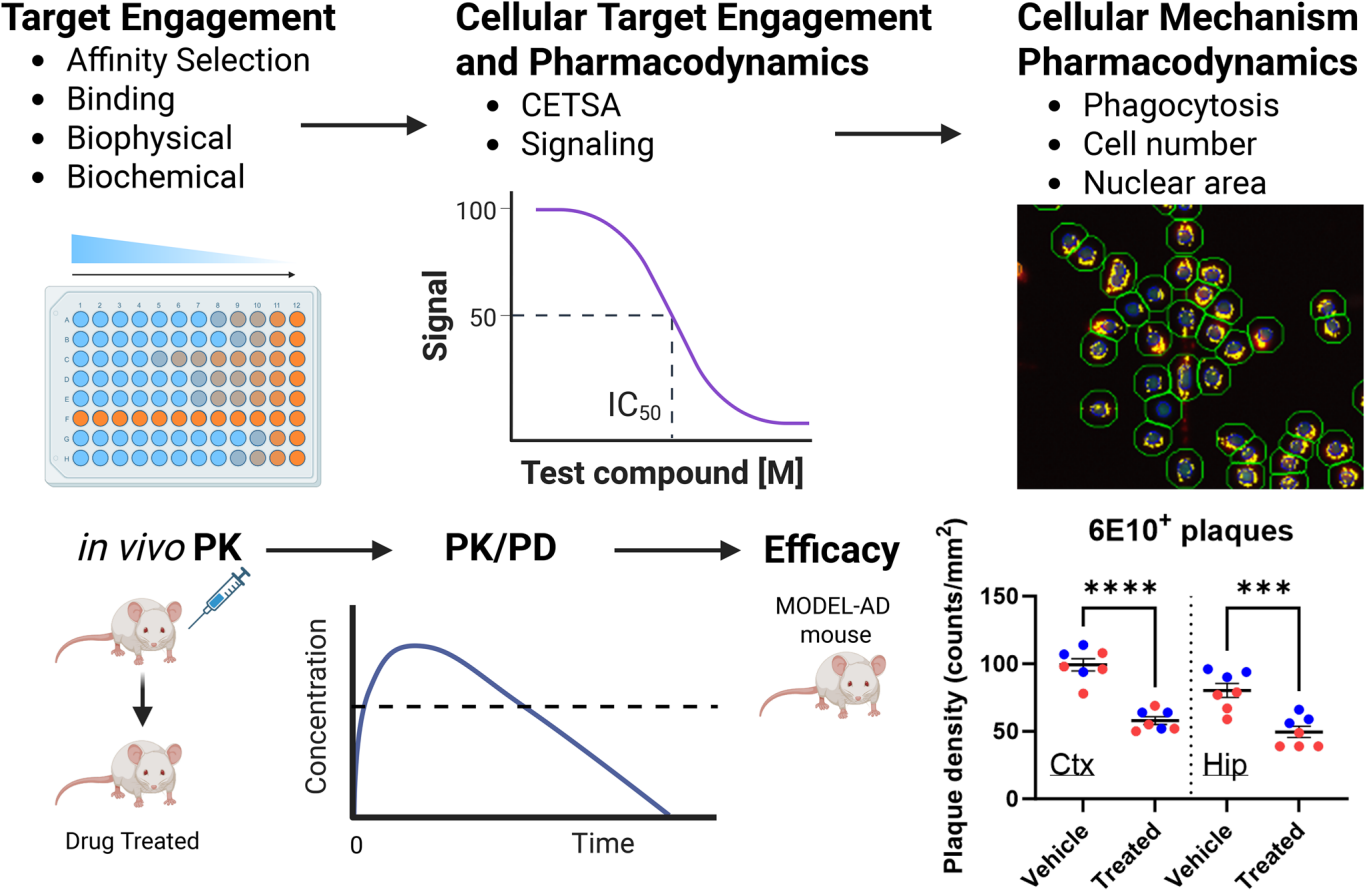
ADHTS Core strategy for a connected flow scheme. Target engagement assays (e.g., affinity, binding, biophysical, biochemical) are correlated to cellular target engagement and pharmacodynamics (CETSA, signaling) assays that are predictive of activity in phenotypic cellular assays (e.g., phagocytosis, cell morphology) and capable of driving SAR studies. These cellular activities are linked to in vivo pharmacokinetics, pharmacodynamics, and efficacy studies, enabling validation of mechanistic hypotheses and advancing targets from tool compound discovery to lead optimization. Created with biorender.com. ADHTS, Assay Development and High Throughput Screening; CETSA^®^ (CEllular Thermal Shift Assay) is a patented technology developed and owned by Pelago Bioscience AB; SAR, structure–activity relationship.

Throughout this process, assays with adequate sensitivity and reproducibility that guide molecular design and SAR studies are evaluated and prioritized with flow scheme connectivity as a critical guide to success. A connected flow scheme is achieved when activity in one assay predicts activity in the following assays, thus providing the ability to advance ligand optimization through iterative learning and to compare chemical scaffolds (Figure 5). Target‐based binding, biophysical, and biochemical assays should predict cellular responses, which in turn predict in vivo PD that correlates to in vivo efficacy with a sufficient probability of accurately projecting human drug exposures and PD responses for the design of high‐quality clinical trials in which translational measures informed by these preclinical studies are used. Successful enablement of in vitro assays and identification of in vivo strategies go hand in hand with the identification or development of quality tool molecules that can be used to study the pharmacology of molecules and connect the assays through the predictive associations described earlier. The delivery of a connected flow scheme with relevant profiling or selectivity assays is the ultimate goal of the ADHTS Core.

### 3.4 Target‐to‐probe molecule

For a molecule to serve as a drug lead candidate, it must possess suitable properties for its intended clinical use. A common pitfall in early‐stage drug discovery campaigns is the prioritization of target potency and selectivity at the expense of desired pharmacology and developability, which are the physicochemical parameters and ADME properties required for in vivo exposures sufficient for TE in human clinical studies. While potency and selectivity are essential elements of a pharmacological tool for target validation, neglecting these other drug‐like properties often leads to dead ends and failure to achieve translational goals. For an orally bioavailable small molecule, this includes adequate physicochemical properties of solubility, permeability, stability in biofluids, and resistance to metabolic clearance in vivo. For an oligonucleotide, established guidelines for sequence selection, nucleotide modification, and conjugation strategies for cellular delivery must be followed. For an antibody, parameters beyond affinity and specificity include ensuring appropriate half‐life and biodistribution, minimizing immunogenicity, and selecting suitable formats and engineering strategies to ensure TE and therapeutic efficacy.

**FIGURE 6:**
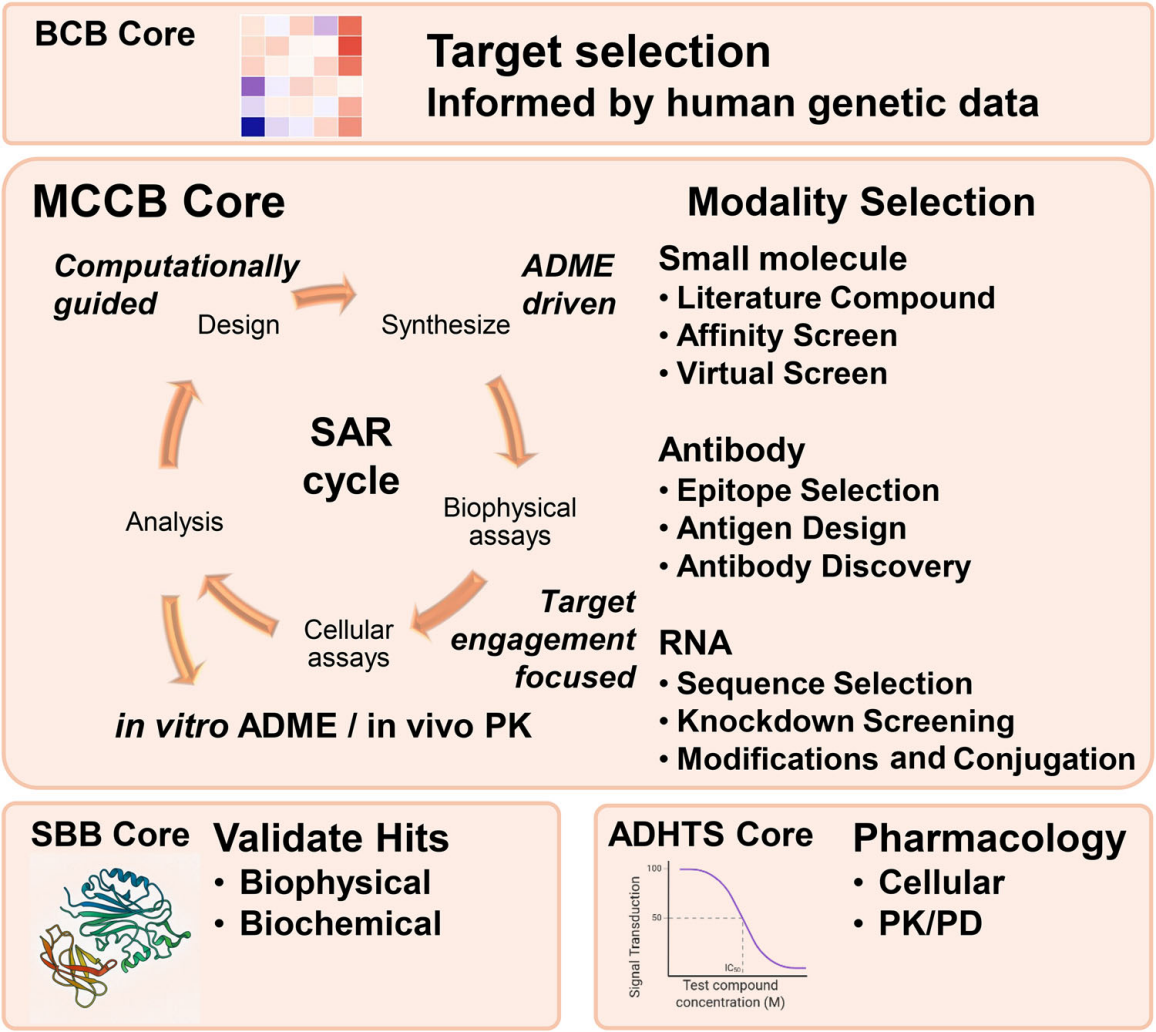
MCCB Core strategy for convergence. Target evaluation guided by the BCB Core drives the selection of the most appropriate therapeutic modality (e.g., small molecule, antibody, and/or RNA‐based therapeutics). Hit validation incorporates biophysical and biochemical assays in collaboration with the SBB Core, while pharmacology and PK/PD studies are coordinated with the ADHTS Core. Iterative SAR cycles, informed by computational design and ADME properties, enable optimization of candidate molecules for lead development. Chemical biology strategies support target engagement and exploration of MOA. ADHTS, Assay Development and High Throughput Screening; ADME, Absorption, Distribution, Metabolism, and Excretion; MCCB, Medicinal Chemistry and Chemical Biology; MOA, mode of action; PD, pharmacodynamics; PK, pharmacokinetics; SAR, structure–activity relationship; SBB, Structural Biology and Biophysics.

A critical early step in our workflow is the selection of an appropriate therapeutic modality, whether small molecule, oligonucleotide, or antibody based on the biological target and intended mechanism of action (Figure 6). Factors influencing this decision include the cellular localization of the protein of interest (e.g., cytosolic enzyme vs transmembrane receptor), the desired modulation (activation vs inhibition), the structural or functional characteristics of the protein (e.g., catalytic kinase or scaffolding protein), and the competitive landscape in the literature. This critical assessment ensures resources are focused on strategies that are clear, rational, and tractable. While our team has experience with all three therapeutic modalities, for the purposes of this paper we will focus further details on the discovery and development of small molecules.

The hallmark of our approach is the early emphasis on direct TE. For small molecules in particular, ambiguity surrounding the mechanism of action is a common challenge. To mitigate this, we prioritize biophysical or biochemical screening methods that provide unambiguous evidence of protein–ligand interaction before pursuing cell‐based assays. When it has been feasible for primary screening, we have employed affinity selection mass spectrometry (ASMS). In cases where ASMS is not amenable to the desired target, we have leveraged our expertise in virtual screening. When only apo structures are available, we have relied on recent advances in predictive modeling to identify potential binding sites and generate plausible ligand poses. The resulting lists of potential hit compounds are filtered to exclude pan‐assay interference compounds (PAINS)and molecules with unacceptable predicted ADME, using computational tools such as Simulations Plus ADMET Predictor. Collaboration with internal drug metabolism and pharmacokinetics (DMPK) subject matter experts greatly enhances the value of these tools.

Once TE is confirmed using cellular protein thermostability assays, we advance compounds into cellular pharmacology assays designed to measure proximal PD readouts. From there we initiate focused SAR studies to define the minimal pharmacophore and identify early structure‐activity trends, all while ensuring predicted ADME properties remain within acceptable bounds. Promising compounds are then evaluated in vitro for solubility, permeability, metabolic stability, and protein binding. These data are used to model expected in vivo exposure and guide PK study design. Our early, integrated focus on physicochemical and ADME properties has enabled the successful identification of central nervous system‐penetrant lead compounds with minimal need for extensive structural adaptation of the potent[Fig alz70964-fig-0007] scaffolds.

We aim to validate a workflow that enables our team and collaborating groups to efficiently discover and optimize tool compounds to probe the pharmacology of the target of interest. This flow scheme emphasizes early and direct measurement of TE, cellular activity, and drug‐like properties. We identify optimized molecules that not only bind to the protein of interest and modulate its pharmacology in a desirable way but also exhibit favorable ADME profiles and sufficient brain exposure for in vivo PD studies. Such tool compounds allow us and the broader research community to not only interrogate the pharmacology of the specific tool compound but also explore the biological relevance of targeting that protein in preclinical models. By combining medicinal chemistry with biophysics and cellular targets early in the discovery process, our core aims to reduce the risk of translational failure and accelerate the path from target nomination to in vivo validation.

## 4 CONCLUSION

The IUSM–Purdue TREAT‐AD Center was established within a broader national effort by the NIH to bridge the gap between newly understood genetic risk factors of AD and therapeutic development. On the basis of output from the AMP‐AD initiative, the center was launched by the NIA with a focus on systems biology and translational approaches to target nomination and enablement for drug discovery. The center leverages deep institutional expertise and longstanding programs in AD research at IUSM. Our collaborative structure with Purdue University, the University of Pittsburgh, and key research partners like the IBRI is designed to advance novel biological targets grounded in human biology into drug lead development programs with cutting‐edge cross‐functional capabilities. With a foundation in open science, the center also shares findings with the global scientific community to build on our research and further advance the field.

The center's strategy is anchored in a milestone‐driven operational model that integrates four technical cores overseen by an administrative core. This multidisciplinary framework ensures rigor at each step of enablement, from initial target nomination and prioritization through target characterization and assay development to chemical probe optimization. Candidate targets are selected and prioritized on the basis of multi‐omics datasets and assessment of biological validation and molecular tractability. Selected targets are enabled by a connected flow scheme of assays for rigorous pharmacological assessments of ligands. The assays are designed and validated to produce reproducible results and demonstrate an ability to distinguish structural changes as part of SAR studies. These strategies are coordinated for iterative evaluation of molecular potency, selectivity, and drug‐like properties, ensuring that promising molecules have a trajectory toward a lead development program.

**FIGURE 7 alz70964-fig-0007:**
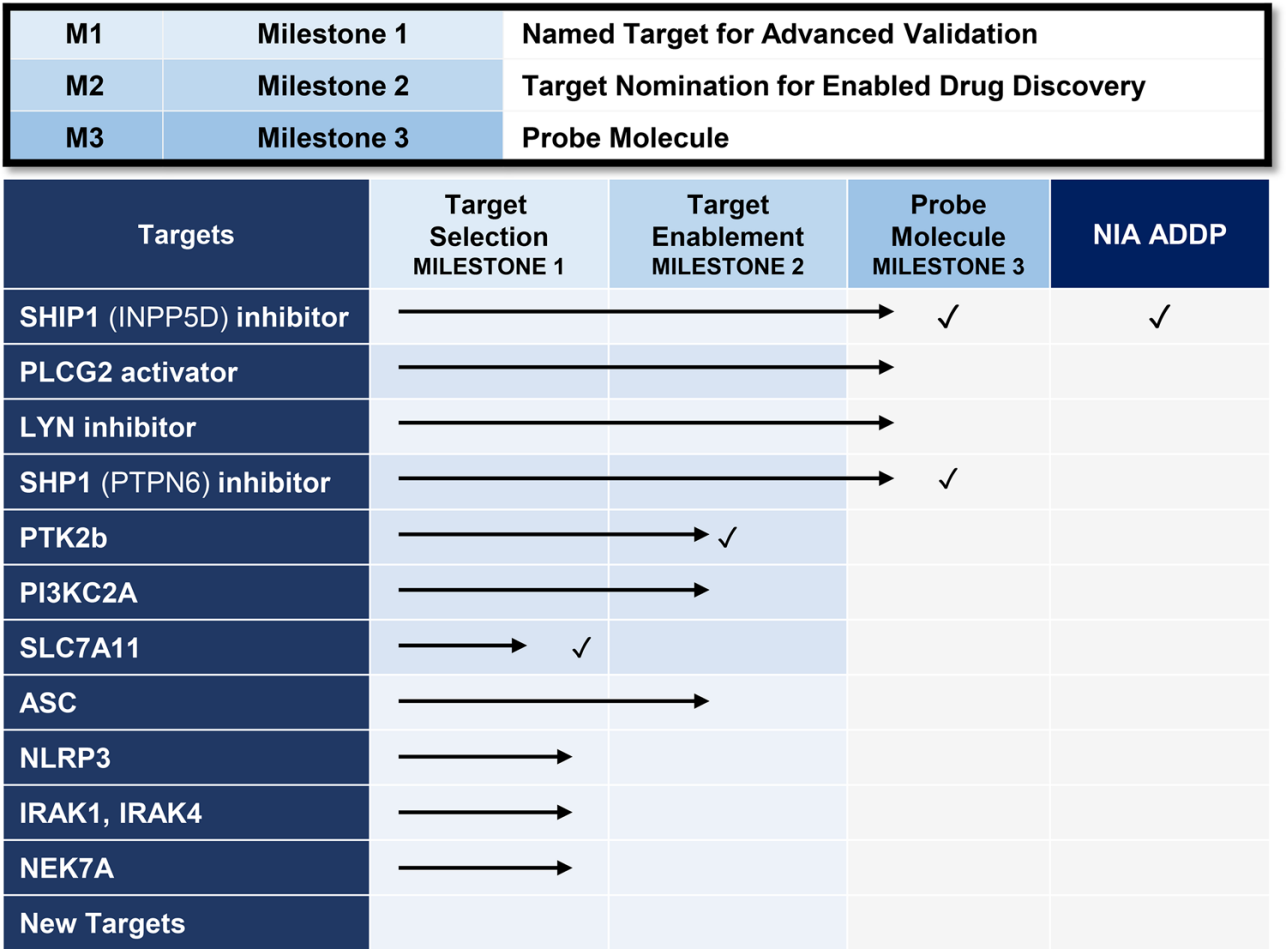
Current portfolio of enabled targets. Each target was evaluated through the milestone progression outlined in Figure 2. Our most advanced project, SHIP1, has transitioned to the ADDP. Other targets remain in progress or were concluded at the milestone stage indicated by a check mark. ADDP, Alzheimer's Drug Development Program.

The TREAT‐AD Centers have taken a target‐based approach rooted in human genetics, systems biology, and mechanistic validation to produce a growing portfolio of targets enabled for drug discovery (Figure 7). Each target in the portfolio is documented by a comprehensive TEP that includes validated biological assays, tool compounds, structural data, and translational biomarkers. These TEPs are openly shared via the AD Knowledge Portal. Although our approach is necessarily target‐centric, it is complementary to phenotypic screening strategies focused on desired cellular or physiologic responses rather than preselected target hypotheses.^19^ Integrating insights from both target‐based and large‐scale phenotypic approaches will ultimately strengthen the pipeline of drugs for complex diseases such as AD. This body of work not only accelerates the pipeline for novel AD therapeutics but also provides a roadmap for how academic centers can integrate multidisciplinary capabilities to de‐risk early‐stage drug discovery. Thus, the TREAT‐AD Center serves as a key catalyst and invaluable partner to global scientists seeking the discovery of next‐generation therapeutics and biomarkers.

## CONFLICT OF INTEREST STATEMENT

TIR, KH, JZ, ADM, JLD, BC, BTL, and ADP are founders and consultants for Monument Biosciences and own company stock. TIR is a consultant for Candenza and has stock options. BCK has stock in Evecxia Therapeutics. TIR and BC have patents planned or pending related to this work. JLD is an inventor on patents or patent applications assigned to Eli Lilly and Company relating to the assays, methods, reagents, and/or compositions of matter for p‐tau assays and Aβ‐targeting therapeutics, has/is served/serving as a consultant or on advisory boards for Eisai, AbbVie, Genotix Biotechnologies Inc., Gates Ventures, Syndeio Biosciences, Dolby Family Ventures, Karuna Therapeutics, Alzheimer's Disease Drug Discovery Foundation, AlzPath Inc., Cognito Therapeutics, Inc., Eli Lilly and Company, Prevail Therapeutics, Neurogen Biomarking, Spear Bio, Rush University, University of Kentucky, Tymora Analytical Operations, MindImmune Therapeutics, Inc., Early Is Good, and Quanterix, has received research support from ADx Neurosciences, Fujirebio, Roche Diagnostics, and Eli Lilly and Company in the past 2 years, has received speaker fees from Eli Lilly and Company and LabCorp, has stock or stock options in Eli Lilly and Company, Genotix Biotechnologies, MindImmune Therapeutics, Inc., AlzPath Inc., and Neurogen Biomarking. BL receives licensing fees from Ionis Pharmaceuticals, participates on the advisory board and receives consulting fees from NervGen Inc. and The Cleveland Clinic, has a leadership or fiduciary role at the Alzheimer's Association and Cure Alzheimer's Fund, has received travel support from Alzheimer's Association, Cure Alzheimer's Fund, and the Department of Defense. ADP is president and CEO of the Indiana Biosciences Research Institute (IBRI). All funding provided to the institution and individual authors has been disclosed in the funding sources. Author disclosures are available in the supporting information.

## Supporting information



Supporting Information
